# Effects of 8-week TRX exercise program on bowel habits and gastrointestinal symptoms in competitive male swimmers aged 9–13: a randomized controlled trial

**DOI:** 10.1186/s13102-026-01806-6

**Published:** 2026-06-15

**Authors:** Yağmur Yıldız, Ayşe Nur Kahve

**Affiliations:** Department of Sports and Health, Aksaray University, Aksaray, 68100 Türkiye

**Keywords:** TRX exercise, Bowel habits, Gastrointestinal symptoms, Swimming

## Abstract

**Background:**

Exercise-related gastrointestinal complaints are frequently reported in young athletes; however, evidence regarding the effects of resistance-based training modalities on bowel habits is limited. The aim of this study was to investigate the effect of an 8-week TRX program on bowel habits and gastrointestinal system of male child swimmers.

**Methods:**

Thirty-two prepubertal male swimmers aged 9–13 years (age: 10.87 ± 1.16 years; height: 148.0 ± 34.0 cm; weight: 40.82 ± 8.3 kg) were voluntarily enrolled in the study. Participants were randomly allocated to either the TRX or control group. Both groups were evaluated using the Bristol Stool Scale and a questionnaire assessing defecation habits before and after the 8-week TRX exercise program.

**Results:**

Descriptively, the proportion of participants who postponed defecation decreased in the TRX group, whereas an opposite trend was observed in the control group. No clear change in stool form was detected between groups. A mixed-design repeated-measures ANOVA revealed a significant group × time interaction for total gastrointestinal symptom scores (F(1,30) = 13.73, *p* = 0.001, partial η²=0.314), indicating that TRX training led to greater improvements compared with the control group.

**Conclusion:**

These findings suggest that TRX training may represent a safe and potentially beneficial exercise approach for improving gastrointestinal comfort in young athletes.

**Clinical trial number:**

NCT07464509-11/02/2026.

**Supplementary Information:**

The online version contains supplementary material available at 10.1186/s13102-026-01806-6.

## Introduction

Gut health is a key determinant of overall well-being and quality of life [1]. Stool characteristics such as consistency, colour and frequency and defecation patterns are widely recognized as important indicators of gastrointestinal and are important indicators of general health [2]. Defecation frequency and habits of an individual in childhood have been associated with environmental factors such as gender, diet, age [3], genetic makeup, parental behavior, social background and cultural beliefs [4]. Although functional bowel problems are quite common in the community, they can be prevented and treated with lifestyle changes such as a diet high in fiber, increased fluid intake and physical activity [5].

Previous studies have shown that regular physical activity may improve bowel function by shortening bowel transit time. Moreover, physical activity levels have also been associated with improved defecation patterns and greater defecation frequency, particularly in individuals with chronic constipation symptoms [6–8]. One of the most popular resistance training methods in recent years is the total body resistance exercise (TRX). TRX suspension training is a form of bodyweight resistance exercise performed under unstable conditions. TRX exercises are very suitable for improving health and athletic performance [9–11] and may potentially influence bowel motility and gastrointestinal function. Previous studies have demonstrated that TRX training can significantly influence metabolic and physiological parameters. However, these studies were conducted with obese, chronic back pain and sedentary individuals [12, 13].

TRX exercises are not merely a form of training that increases overall physical activity levels; they are also a functional resistance training modality that demands intensive core stabilization, multi-joint muscle activation, and postural control [9]. Owing to these characteristics, TRX may exert indirect effects on the gastrointestinal system; in particular, activation of the abdominal and pelvic floor muscles is thought to influence bowel function through the regulation of intra-abdominal pressure and autonomic nervous system responses that can affect intestinal motility. In this study, TRX exercises are performed in an unstable environment and require continuous core activation, distinguishing them from traditional exercise modalities [14, 15]. This condition aligns with mechanisms reported in the literature that support the effects of physical activity on intestinal transit time and defecation frequency [6, 16].

In the literature, physical activity has been shown to have beneficial effects on bowel habits across different populations [6, 17, 18]. However, the effects of specific and contemporary exercise modalities, such as TRX suspension training, on gastrointestinal function and bowel habits remain insufficiently explored [19, 20]. Although TRX training has been widely investigated in terms of performance and metabolic outcomes, its potential impact on gastrointestinal health has not been adequately examined. Moreover, existing studies predominantly focus on adult or clinical populations, while evidence in pediatric athletic populations is notably limited. To obtain a relatively homogeneous sample with respect to age, training background, and physiological characteristics, male swimmers aged 9–13 years were recruited for this study. Bowel habits and gastrointestinal symptoms are known to be influenced by a variety of factors, including age, sex, environmental conditions, and physical activity levels [3, 6]. Therefore, restricting participation to male swimmers was intended to minimize the influence of potential confounding variables and enhance the internal validity of the study. In addition, swimmers constitute an appropriate athletic population for investigating the effects of a TRX training intervention, as their engagement in regular and structured training programs enables a more controlled assessment of intervention-related outcomes. Although the relationship between physical activity and gastrointestinal function has been extensively investigated, existing evidence has predominantly been derived from adult populations [17, 18]. In contrast, studies involving pediatric and adolescent athletes remain scarce. Consequently, the present study addresses an important gap in the literature by focusing on young male swimmers and may provide novel insights into exercise-induced gastrointestinal adaptations during a critical stage of growth and athletic development.

Therefore, the present study aimed to investigate the effects of an 8-week TRX training program on bowel habits and gastrointestinal symptoms in male child swimmers aged 9–13 years, addressing an important gap in the current literature. We hypothesized that TRX exercise would lead to a significant improvement in total gastrointestinal symptom scores in male swimmers aged 9–13 years.

## Methods

### Participants

Thirty-two male children who were swimmers (age: 10.87 ± 1.16 years; height: 148.0 ± 4.3 cm; body mass: 40.82 ± 8.3 kg; BMI: 18.64 kg/m²) voluntarily participated in the study. Sample size was determined using a priori power analysis based on a repeated-measures ANOVA (within–between interaction) design. Calculations were performed using G*Power (version 3.1.9.6) with an effect size f of 0.15, an alpha error probability of 0.05, a statistical power (1 − β) of 0.80, two groups, and two measurement points (pre–post). The required total sample size was calculated as 30 participants. Considering possible dropouts, the sample size was increased to 32 participants. Male swimmers between the ages of 9–13 years who have been performance athletes for at least 2 years, athletes who practice swimming at least 3 days a week, athletes who have not participated in any resistance training other than normal swimming training in the last six months, and athletes who do not have chronic diseases such as asthma and chronic heart diseases were included in the study. Athletes with chronic diseases such as asthma and chronic heart diseases, non-performance swimmers, athletes who participated in any resistance training other than normal swimming training in the last six months, athletes whose parental consent forms were not obtained, athletes who did not participate in more than 3 training sessions and athletes who gave misleading answers or did not answer the questionnaire questions properly were not included in the study. No adverse event occurred during the study. Before the commencement of the study, as the athletes were under 16 years of age, their parents were informed and written informed consent was obtained from the parents. Ethical approval for the study was granted by the Aksaray University Ethics Committee (Decision No: 2025/72), and the study was conducted in accordance with the principles of the Declaration of Helsinki. This study was registered at ClinicalTrials.gov (Identifier: NCT07464509) on February 11, 2026.

### Experimental design

**Fig. 1:**
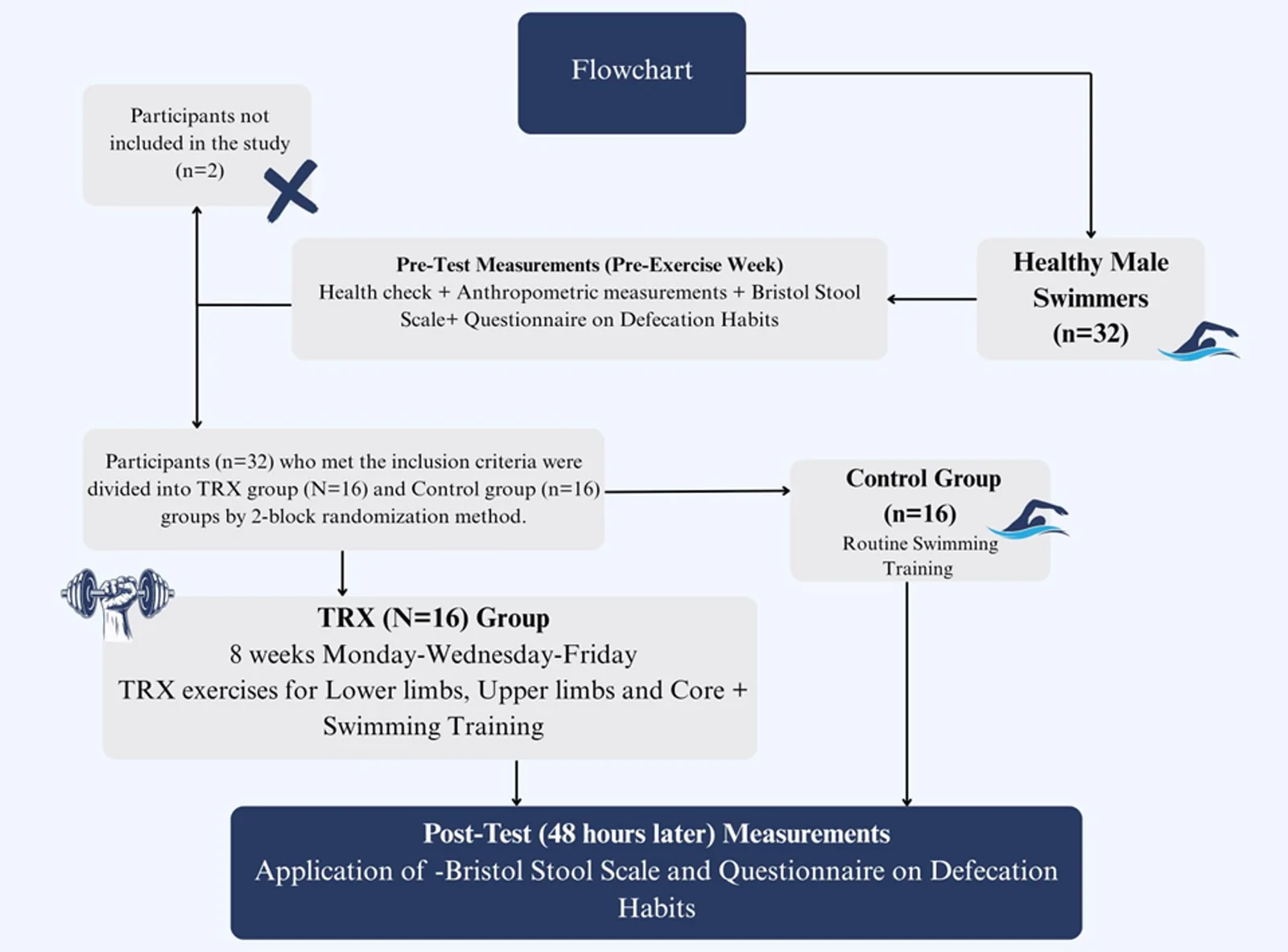
Flowchart of study design

This study was conducted as an 8-week randomized controlled trial with 32 male swimmers. Participants were randomly assigned to either the TRX group or the control group using a computer-generated randomization sequence (Random Allocation Software). The allocation process was conducted by a researcher who was not involved in data collection or outcome assessment to minimize potential selection bias. After the pre-test assessments, one participant in the experimental group was excluded due to incomplete training, and one participant in the control group was excluded due to non-participation in post-test assessments. The TRX group and the control group were administered the Bristol Stool Scale and a questionnaire assessing defecation habits before and after the 8-week TRX exercise program (Fig. 1).

Before the 8-week TRX training program, a familiarization (adaptation) session was applied to the TRX group. The purpose of this session was to ensure that participants became familiar with the TRX exercises, to control early performance changes related to motor learning, to standardize exercise techniques, and to enhance the internal validity of the measurements related to the intervention. In addition, reducing the potential risk of injury associated with unstable exercises was among the objectives of the familiarization session. Since this study aimed to compare the effect of TRX exercise model with constipation and bowel habits, no additional food (apricots, plums, etc.) and medication (laxatives, etc.) were given to the children.

During the study period, all training sessions were performed between 12.00 and 13.30 (± 1 h), Monday-Wednesday-Friday. The athletes participated in a total of 24 exercise sessions 3 days a week for 8 weeks. Exercise intensity was determined according to TRX stability, vectorial and pendulum principles. Exercise intensity was determined based on the level of instability created by the TRX suspension system. During the movements, the stability level was increased or decreased by altering the body’s position relative to its center of gravity (stability principle). In addition, the difficulty of the exercises was adjusted by changing the body angle, which modified the direction and magnitude of the applied force (vectorial loading), as well as through balance control induced by the oscillatory movement of the suspension straps (pendulum effect). In this way, the level of loading experienced by the participants during exercise was systematically and controllably manipulated. Exercise was monitored using the Angle Meter 360 and the My Jump application. Mobile applications show the angle of the squat exercise so the examiner can track the position of the swimmer and check the range of motion by using an iOS-based mobile phone. The exercise protocol, questionnaire and scales were administered in a gym at a temperature of 20–24 °C.

During the study period, participants did not participate in any swimming competitions, and the study was conducted during the summer break period. A total of 10 exercises were applied to the participants. No additional swimming training sessions were implemented during the intervention period outside the study protocol. Participants were instructed to adhere only to the prescribed training program and were informed not to engage in any additional exercise or training activities beyond the study protocol. Since the study was conducted during the summer holiday period, participants did not have any participation in additional physical activities such as physical education classes.

These exercises consisted of movements for the upper body, lower body and core. Core exercises include Bicycle Crunch and Knee Tuck; lower extremity exercises include Calf Rise, Hamstring Curl, Lateral Skater With Stick and TRX Squat Jump; and finally upper extremity exercises include Chest Fly, Chest Press, Biceps Curl and TRX Inverted Row. Swimmers were given 45 s rest time between sets and 2 min rest time between each exercise. Core exercises (Bicycle Crunch, Knee Tuck) were selected to improve trunk stability, which is critical for maintaining body position in swimming and ensuring force transfer between the extremities. Lower extremity exercises (Calf Raise, Hamstring Curl, Lateral Skater with Stick, TRX Squat Jump) were programmed to support propulsion force, balance, unilateral control, and the development of explosive strength. Upper extremity exercises (Chest Fly, Chest Press, Biceps Curl, TRX Inverted Row) were applied to enhance push-pull strength balance contributing to swimming propulsion, as well as shoulder girdle stability [21, 22]. The Bristol stool scale and questionnaires including defecation habits were administered before starting the exercise program and after the 8th week of the training program was completed. No side effects or injuries were observed during the training. This randomized controlled trial was conducted and reported in accordance with the CONSORT statement. The participant flow diagram is presented in Fig. 1.

### Data collection tools

*Bristol Stool Form Scale (BSFS);* was developed by Lewis and Heaton at the University of Bristol to assess the colonic rates of individuals [23]. The shape of the feces differs according to the duration of their stay in the colon. For this reason, BSFS has been recognized as a reliable and rapid indicator of transit time. According to this scale, individuals’ stools are classified into 7 groups, with Form 1 and Form 2 representing slow passage of stool through the colon, Form 3 and Form 4 representing normal passage of stool through the colon, and Form 5 and Form 6 representing rapid passage of stool through the colon and impaired rectal sensitivity [24].

### Statistical analysis

All statistical analyses were performed using IBM SPSS Statistics version 30.0 [25]. Descriptive statistics are presented as mean ± standard deviation for continuous variables and as number (n) and percentage (%) for categorical variables. The normality of continuous variables was assessed using the Shapiro–Wilk test. As gastrointestinal symptom scores did not meet the assumption of normality, log transformation was applied prior to analysis.

The primary outcome of the study was total gastrointestinal symptom score. To examine the effects of the intervention, a mixed-design repeated-measures analysis of variance (ANOVA) was conducted with one between-subject factor (group: TRX vs. control) and one within-subject factor (time: pre-test vs. post-test). The group × time interaction term was used to evaluate the differential effect of the intervention between groups. Effect sizes were reported as partial eta squared (η²). Statistical significance was set at *p* < 0.05.

## Results

The bowel habits of the 32 male swimmers included in the study are given in Table 1.

**Table 1:** Bowel habits of the participants

		TRX group (*n* = 16)		Control group (*n* = 16)	
		Pre test *n* (%)	Post test *n* (%)	Pre test *n* (%)	Post test *n* (%)
Habit of delaying bowel movements	I postpone	9 (56.2)	3 (18.7)	9(56.2)	11 (68.7)
	I don’t postpone	7 (43.8)	13 (81.3)	7(43.8)	5 (31.3)
Reasons for delaying bowel movements	Avoiding the use of public toilets	9 (100.0)	-	5 (55.6)	7 (63.6)
	Lack of appropriate opportunity	-	-	-	1 (9.1)
	Lack of urge to defecate	-	-	-	-
	Non-compliant	-	-	4 (44.4)	3 (27.3)
	Other	-	-	-	-
Defecation frequency	Once daily	2 (12.5)	2 (12.5)	4(25.0)	9 (56.3)
	2–3 times daily	9 (56.2)	13 (81.2)	8 (50.0)	4 (25.0)
	Once every two days	4 (25.0)	1 (6.3)	1 (6.3)	-
	Once every three days	1 (6.3)	-	3 (18.7)	3 (18.7)
Time of defecation	Morning	-	-	5 (31.1)	6 (37.5)
	Noon	3 (18.8)	1(6.3)	1 (6.3)	2 (12.5)
	Afternoon	2 (12.5)	1(6.3)	1 (6.3)	1 (6.3)
	Evening	1 (6.3)	1(6.3)	1 (6.3)	2 (12.5)
	Morning-Evening	6 (37.5)	5 (31.3)	8 (50.0)	4 (25.0)
	Morning-Noon-Evening	4 (25.0)	8 (50.0)	-	1 (6.3)

The bowel habits of the 32 male swimmers are presented in Table 1. Descriptively, the proportion of participants who postponed defecation decreased in the TRX group (from 56.2% to 18.7%), whereas an increase was observed in the control group (from 56.2% to 68.7%). Similarly, the proportion of participants reporting defecation 2–3 times per day increased in the TRX group (from 56.2% to 81.2%), while it decreased in the control group. Changes in the time of defecation also suggested a more regular pattern in the TRX group following the intervention.

The distribution of Bristol Stool Form Scale (BSFS) categories is shown in Table 2. The proportion of participants classified as Type 3 and Type 4 (indicative of normal intestinal transit) increased from 81.2% to 87.5% in the TRX group. However, no clear pattern suggesting a substantial shift in stool form was observed between the groups.

**Table 2:** Classification of participants according to the Bristol Stool Form Scale (BSFS)

Variables		TRX group (*n* = 16)		Control group (*n* = 16)	
		Pre test *n* (%)	Post test *n* (%)	Pre test *n* (%)	Post test *n* (%)
Bristol Stool Scale	Type 1	-	-	-	-
	Type 2	1 (6.3)	-	2 (12.5)	-
	Type 3	4 (25.0)	1 (6.3)	4 (25.0)	7 (43.8)
	Type 4	9 (56.2)	13 (81.2)	7 (43.7)	6 (37.5)
	Type 5	2 (12.5)	2 (12.5)	3 (18.8)	2 (12.5)
	Type 6	-	-	-	1 (6.3)

Repeated-measures ANOVA revealed a significant main effect of time (F(1,30) = 27.54, *p* < 0.001, partial η²=0.48) and a significant group × time interaction (F(1,30) = 13.73, *p* = 0.001, partial η²=0.31), indicating that changes in gastrointestinal symptom scores over time differed significantly between the TRX and control groups (Table 3).

**Table 3:** Effects of TRX training on total gastrointestinal symptom scores

Group	Pre test Mean ± SD	Post test Mean ± SD	Group × Time Effect F (*p*, η²)
TRX (*n* = 16)	0.72 ± 0.19	0.42 ± 0.22	13.73 (0.001; 0.314)
Control (*n* = 16)	0.56 ± 0.28	0.51 ± 0.30	

Values are presented as log-transformed means ± standard deviations

Participants’ toilet habits are summarized in Table 4. Descriptively, the proportion of participants reporting no pain during defecation increased in the TRX group (from 62.5% to 93.7%), whereas no change was observed in the control group. Additionally, the proportion of participants reporting difficulty in defecation despite urge decreased in the TRX group, suggesting a potential improvement in bowel-related behaviors. No meaningful changes were observed in toilet-related anxiety or discomfort between the groups.

**Table 4:** Participants’ toilet habits

		TRX group (*n* = 16)		Control group (*n* = 16)	
		Pre test *n* (%)	Post test *n* (%)	Pre test *n* (%)	Post test *n* (%)
Are you satisfied with the frequency of your bowel movements?	Never	**-**	**-**	**-**	**-**
	Rarely	-	-	-	-
	Sometimes	1 (6.3)	-	1 (6.3)	-
	Mostly	2 (12.5)	-	4 (25.0)	6 (37.5)
	Always	13 (81.2)	16 (100.0)	11 (68.7)	10 (62.5)
Are you satisfied with the regularity of your bowel movements?	Never	-	-	-	-
	Rarely	-	-	-	-
	Sometimes	1 (6.3)	-	1 (6.3)	-
	Mostly	2 (12.5)	-	1 (6.3)	4 (25.0)
	Always	13 (81.2)	16 (100.0)	14 (87.4)	12 (75.0)
Feeling the urge to defecate but being unable to do so	Never	6 (37.5)	11 (68.8)	9 (56.3)	13 (81.2)
	Rarely	4 (25.0)	3 (18.7)	3 (18.7)	3 (18.8)
	Sometimes	6 (37.5)	-	4 (25.0)	-
	Mostly	-	2 (12.5)	-	-
	Always	-	-	-	-
Experiencing pain during defecation	Never	10 (62.5)	15 (93.7)	13 (81.2)	13 (81.2)
	Rarely	4 (25.0)	1 (6.3)	3 (18.8)	3 (18.8)
	Sometimes	2 (12.5)	-	-	-
	Mostly	-	-	-	-
	Always	-	-	-	-
Have you experienced anxiety due to uncertainty about when you will be able to use the toilet?	Never	14 (87.4)	14 (87.5)	15 (93.7)	14 (87.5)
	Rarely	1(6.3)	2 (12.5)	1 (6.3)	2 (12.5)
	Sometimes	1 (6.3)	-	-	-
	Mostly	-	-	-	-
	Always	-	-	-	-
Have you experienced increasing discomfort due to being unable to use the toilet?	Never	14 (87.4)	15 (93.7)	14 (87.5)	12 (75.0)
	Rarely	1 (6.3)	1 (6.3)	2 (12.5)	4 (25.0)
	Sometimes	1 (6.3)	-	-	-
	Mostly	-	-	-	-
	Always	-	-	-	-

## Discussion

This study investigated the effects of an 8-week TRX exercise program on bowel habits and gastrointestinal symptoms in young male swimmers. The main findings indicate that TRX training led to a significant improvement in total gastrointestinal symptom scores. In addition, descriptively, a reduction in defecation delay behavior was observed in the TRX group. These findings suggest that TRX exercise may contribute to improvements in bowel-related behaviors and gastrointestinal comfort in male swimmers aged 9–13 years.

The relationship between bowel habits and physical activity has been widely investigated, with evidence suggesting that even light physical activity, compared with rest, is associated with increased colonic motility and facilitated defecation [18]. As presented in Table 1, swimmers in the TRX group showed a reduction in the tendency to postpone defecation, on the other hand the control group demonstrated an opposite pattern over the study duration. Delaying defecation is a common behavior, particularly in situations where individuals avoid using public toilets [26]. Such behavior may lead to irregular bowel habits and contribute to constipation over time [27]. In this context, the observed reduction in defecation postponement in the TRX group may reflect an improvement in bowel-related behaviors. Additionally, in the present study, postponement of defecation was primarily attributed to reluctance to use public toilets, highlighting the potential influence of environmental factors on bowel habits (Table 1).

Experimental studies have shown that voluntary postponement of defecation may reduce colonic transit and lead to irregular bowel habits [28, 29]. In the present study, as presented in Table 2, no clear change in stool form distribution was observed following the TRX intervention. In particular, the proportion of participants classified within normal stool types (Type 3 and Type 4) showed only a slight increase in the TRX group, without indicating a substantial shift in stool characteristics.

These findings do not suggest that physical activity is ineffective in relation to bowel function. On the contrary, previous studies have demonstrated that regular physical activity may improve gastrointestinal motility and reduce the risk of constipation [27, 30, 31]. However, the effects of exercise on gastrointestinal outcomes may vary depending on factors such as exercise intensity, duration, and dietary intake [31]. Therefore, the absence of a clear change in stool form in the present study may be related to these factors. Additionally, it should be considered that stool form is not always a direct indicator of colonic transit, and may be influenced by multiple physiological and behavioral variables [32]. In recent years, research has highlighted both positive and negative effects of physical activity on gastrointestinal function. While moderate exercise is generally associated with improved gastrointestinal health [33–37], high-intensity or prolonged exercise may lead to gastrointestinal disturbances in athletes [38–41].

The present findings, as summarized in Table 3, demonstrate a significant group × time interaction for total gastrointestinal (GI) symptom scores following an 8-week TRX exercise intervention. Specifically, participants in the TRX group showed a marked reduction in log-transformed GI symptom scores from pre- to post-training, whereas the control group exhibited minimal change. The reduction in GI symptom scores may have practical implications for daily comfort, psychological well-being, or athletic performance in male swimmers aged 9–13 years. The significant interaction effect (F(1,30) = 13.73, *p* = 0.001, partial η² = 0.314) indicates that TRX exercise elicited greater improvements in overall GI symptoms compared with continued standard swimming training alone. These results align with a growing body of evidence suggesting that regular moderate physical activity exerts beneficial effects on gastrointestinal function [42, 43]. Studies have reported that regular moderate exercise is associated with increased bowel motility and consequently reduced constipation, leading to improved overall digestive comfort, likely due to increased blood flow, activation of smooth muscle peristalsis, and modulation of neuroendocrine signals regulating bowel function (e.g., improved vagal tone and reduced systemic inflammation) [44, 45]. In contrast, acute or high-intensity exercise has generally been associated with temporary gastrointestinal discomfort, including symptoms such as nausea and abdominal cramps, by causing decreased splanchnic blood flow and mechanical stress on the gastrointestinal system [45]. The findings of this study are consistent with this literature, but demonstrate that a structured, resistance-based exercise program like TRX, when performed at moderate intensity under supervision, may lead to improvements rather than negative gastrointestinal effects. The controlled and professional application of TRX exercise may help explain the observed reduction in gastrointestinal symptoms. In addition to the literature on bowel motility and transit time, the current findings can also be interpreted in terms of neuromuscular control and postural regulation. TRX training emphasizes trunk stabilization, controlled breathing, and coordinated movement patterns, which can indirectly influence intra-abdominal pressure dynamics and visceral support [46, 47]. Improved trunk stability and more effective diaphragm function can contribute to more efficient coordination between the abdominal wall and pelvic floor, facilitating bowel emptying and reducing feelings of bloating or discomfort. Another point to consider is the potential psychosomatic dimension of gastrointestinal symptoms in young athletes. Functional gastrointestinal complaints are known to be sensitive to stress, arousal, and autonomic balance [48]. TRX training, when performed in a structured and controlled environment, can increase body awareness, perceived physical competence, and enjoyment of exercise, thus indirectly regulating stress-related gut-brain signaling. This pathway may also explain improvements in gastrointestinal symptoms observed without an accompanying increase in exercise volume or intensity. Therefore, these findings provide data on how exercise type and context affect digestive health, highlighting the potential benefit of incorporating TRX training into routines that focus not only on performance but also on gastrointestinal well-being.

In the TRX group, the proportion of participants reporting no pain during defecation increased from 62.5% at baseline to 93.7% following the intervention (Table 4). This descriptive improvement may suggest a beneficial effect of TRX exercise on bowel-related comfort. Previous studies have also indicated that regular physical activity may contribute to reduced pain during defecation and improvements in constipation-related symptoms [44, 49, 50].

However, these findings should be interpreted with caution, as pain during defecation may also be influenced by other factors, including underlying medical conditions and individual variability.

## Limitations

Several limitations should be considered when interpreting the findings of this study. First, dietary intake and hydration factors, including fiber consumption and fluid intake, were not controlled or monitored, although these are known to influence gastrointestinal function. Therefore, their potential confounding effects cannot be excluded. The absence of monitoring of dietary intake, fluid intake, sleep patterns, and stress levels represents a limitation of our study. Second, although the participants were selected to be homogeneous in terms of age, health status, gender, and athletic background, the relatively small sample size and the inclusion of only male child swimmers limit the generalizability of the findings. Third, gastrointestinal outcomes were assessed using self-reported measures, which may be subject to reporting bias. In addition, the absence of objective indicators such as gastrointestinal transit time or physiological biomarkers may limit the precision and strength of the findings. In addition, although medication use was assessed and participants using medication were excluded, other unmeasured factors may have influenced gastrointestinal responses. Despite these limitations, this study provides novel evidence regarding the effects of TRX training on gastrointestinal outcomes in pediatric athletes. Future studies should incorporate detailed dietary assessment, hydration monitoring, and objective physiological measures to better understand the mechanisms underlying these effects. 

## Conclusions

In conclusion, the present study demonstrates that an 8-week TRX exercise program may contribute to improvements in gastrointestinal outcomes in male child swimmers. TRX training was associated with a significant reduction in total gastrointestinal symptom scores, as indicated by the group×time interaction in the mixed-design ANOVA. Descriptively, a decrease in defecation postponement and improvements in certain bowel-related behaviors were observed in the TRX group, while no clear change was detected in stool form. These findings suggest that structured, moderate-intensity resistance-based exercise such as TRX may enhance gastrointestinal comfort without inducing adverse gastrointestinal effects in pediatric athletic populations. However, these results should be interpreted with caution, as dietary factors and hydration status were not controlled. Further randomized controlled trials incorporating detailed dietary assessment and objective gastrointestinal measures are warranted to confirm these findings. Finally, future studies should investigate the effects of TRX exercise on gastrointestinal outcomes using larger and more heterogeneous samples, including female athletes and participants from different sport disciplines.

## Supplementary Information

Supplementary Material 1

## Data Availability

The data that support the findings of this study are available from the corresponding author upon reasonable request.
